# Tiny but powerful: protoplast isolation in *Solanum melongena* L. and RNPs mediated genome editing

**DOI:** 10.3389/fpls.2026.1846175

**Published:** 2026-07-16

**Authors:** Martina Ferrero, Matías Nicolás González, Irene Perrone, Per Hofvander, Mariette Andersson, Andrea Moglia

**Affiliations:** 1 Department of Agricultural, Forest and Food Sciences (DISAFA), University of Torino, Grugliasco, Italy; 2 Department of Plant Breeding, Swedish University of Agricultural Sciences, Lomma, Sweden; 3 Institute for Sustainable Plant Protection, National Research Council (CNR-IPSP), Torino, Italy

**Keywords:** CRISPR/Cas9, genome editing, lipofection, protoplasts, ribonucleoproteins, *Solanum melongena* L.

## Abstract

Traditional genetic transformation approaches relying on *Agrobacterium tumefaciens* for the delivery of CRISPR/Cas9 reagents usually provide plants that stably integrate the gene construct in their genome. To meet the EU commission’s proposal for a new legislation on plants obtained by new genomic techniques (NGTs), it is important to develop new protocols that produce transgene-free genome edited plants (NGT category 1). Protoplasts are a promising platform, since delivery of CRISPR/Cas9 reagents as ribonucleoproteins (RNPs) is effective in cells lacking their wall. This allows genetic modifications from a transient application, leaving no traces in the recipient genome apart from the desired targeted mutations. With the aim of implementing transgene-free editing of eggplant (*Solanum melongena* L.), we adapted and improved a protocol previously established in potato and tomato for the isolation of protoplasts from cotyledonary leaves and subsequent CRISPR/Cas9 reagents delivery. Isolated protoplasts were subjected to *in vitro* culture and regeneration, and the first shoots were regenerated from calli approximately 4–5 months after isolation. Alongside, two transfection protocols were tested for the delivery of RNPs into eggplant protoplasts, one using polyethylene glycol (PEG) in two concentrations (25% and 40%) and one exploiting two formulations of lipofectamines (Lipofectamine CRISPRMAX™ and Lipofectamine™ 3000), all targeting *SmChl_H* gene, whose inactivation can cause a chlorotic phenotype. Efficient callus regeneration from transfected protoplasts was obtained and the editing efficiency (calculated as the percentage of edited calli on the total of calli that underwent sequencing) was evaluated. 25% PEG treatment provided the highest editing efficiency, and fully edited biallelic calli were retrieved, showing the expected chlorotic phenotype. Even if the efficiency of *in vitro* regeneration of plants from calli still needs improvement, edited plants were regenerated from protoplasts, representing the first report of RNP mediated genome editing in eggplant protoplasts.

## Introduction

Over the last decades, biotechnological tools have been largely developed with the purpose of deciphering gene function and regulation and for accelerating crop breeding programs. These strategies may take advantage of two main features: the ability to precisely modify genomes and to regenerate a whole plant from one or a few cells.

Since the advent of CRISPR/Cas9 and related gene editing technologies, targeted modification of crop genomes has become easier, more flexible and more accessible, opening avenues for the so-called New Genomic Techniques (NGTs). These strategies most commonly rely on the insertion of CRISPR/Cas9-coding sequences into a host genome followed by segregation of the cassette through selfing, in order to obtain a transgene-free progeny. Alternatively, approaches based on transient applications of CRISPR/Cas9 reagents avoid integration of foreign DNA into the recipient genome (Reed and Bargmann, 2021).

Plant tissue culture is a crucial tool for plant biotechnology: *in vitro* regeneration protocols have been developed for a large number of plant species over the years, exploiting totipotency of plant cells to produce whole organisms from a small amount or even single cells (Levengood et al., 2024).

Despite being part of the well-studied *Solanaceae* family, eggplant (*Solanum melongena* L.) is not keeping pace with biotechnological development if compared to its relative species. In fact, only few successful applications of the CRISPR/Cas9 system have been reported in this species (Ferrero et al., 2024; Kodackattumannil et al., 2023; Maioli et al., 2020; Phad et al., 2024), all of these relying on *Agrobacterium tumefaciens* mediated transformation. On the contrary, many examples can be retrieved from the literature regarding CRISPR/Cas mediated genome editing in other solanaceous crops, such as tomato and potato (Anand et al., 2025; Das et al., 2023; Ferrero et al., 2026; Sapakhova et al., 2025). This difference can be due to both difficulties in obtaining efficient editing and to genotype-dependent recalcitrance to *in vitro* regeneration in eggplant (García-Fortea et al., 2020).

As an alternative to stable genetic transformation, transient protocols (i.e. not involving integration of any exogenous DNA in the genome) may be employed to obtain “transgene free” edited plants that do not require time consuming steps of back-crossing or selfing to eliminate integrated sequences. These protocols usually rely on direct delivery of CRISPR components into protoplasts as either DNA or ribonucleoproteins (RNPs, complexes formed by the Cas9 protein and the guide RNA molecule) (Jiang et al., 2021). RNPs are non-replicative complexes that are degraded rapidly in the cell, ensuring no traces other than the desired genomic modification.

Protoplasts are naked cells derived from the removal of the cell wall through enzymatic digestion. This kind of cells are more prone to introduction of exogenous material because of the absence of the cell wall barrier, and this transfer can be mediated by either electroporation, polyethylene glycol (PEG), or nanoparticles.

Electroporation relies on an electrical field that produces temporary pores in the plasma membrane, allowing the passage of micro- or macromolecules in and out of the cells (Ozyigit, 2020). This technique has been originally employed to support the delivery of CRISPR reagents into animal cells and later applied to some plant species, such as cabbage (Lee et al., 2020) and soybean (Subburaj and Agapito-Tenfen, 2023).

PEG-mediated transfection of RNPs is probably the most common protocol for genome editing in plant protoplasts. Polyethylene glycol destabilizes the plasma membrane of the protoplast, making it permeable to exogenous material, such as RNPs. Many examples of the employment of this technique have been reported in plant protoplasts, such as Arabidopsis (Woo et al., 2015), rice (Woo et al., 2015), apple (Malnoy et al., 2016), potato (Andersson et al., 2018), cabbage (Lee et al., 2020), banana (S. Wu et al., 2020), chili pepper (Kim et al., 2020), maize (Sant’Ana et al., 2020), soybean (Seol et al., 2022), tomato (Y. Liu et al., 2022; Nicolia et al., 2021a), European chestnut (Pavese et al., 2022), wheat (Poddar et al., 2023) and grapevine (Najafi et al., 2023; Osakabe et al., 2018).

Nanoparticles, as an alternative, are artificially made particles with a dimension <100 nm, variable composition and physical/chemical properties that allow interaction with biological molecules (K. Wu et al., 2023). These nanoparticles can be internalized into the plant cells, bringing with them the CRISPR/Cas machinery. In particular, particle bombardment of cells, using metal nanoparticles coated with editing reagents, has been utilized to deliver RNPs into rice protoplasts (Banakar et al., 2020), but other techniques exploiting different nanoparticles and delivery methods can be found (Osmani et al., 2024), for example in maize (Nagy et al., 2023).

An emerging alternative to these three methodologies is represented by lipofection. This approach exploits the ability of cationic lipid to alter the charge of RNPs to positive so that the binding with negatively charged surfaces of the cell is promoted, resulting in uptake and intracellular distribution of the complex (Ghogare et al., 2021). This method has been optimised in animal cell systems (Sousa et al., 2022; Yu et al., 2016) and successfully applied in lettuce (J. Park et al., 2019), tobacco (W. Liu et al., 2020) and citrus (Mahmoud et al., 2022) protoplasts to obtain target genome editing. Moreover, Gambino et al. (2024) demonstrated that PEG-mediated transfection causes cytotoxicity on grapevine protoplasts, while lipofectamine-mediated delivery of editing reagents has a lower impact on the protoplast viability. This allowed *in vitro* regeneration even from the recalcitrant cultivar Nebbiolo, making this approach a promising alternative to traditional transformation protocols.

Within the *Solanum* genus, most reports including the application of CRISPR/Cas9 exploit stable gene transfer methods mediated by *Agrobacterium tumefaciens*, but there are multiple advantages in using RNPs with protoplasts: absence of transgene insertions (DNA-free approach), rapid degradation of RNPs leading to low off-target effects and chimerism, and the elimination of time consuming cloning steps to prepare transformation vectors to insert T-DNA in the target plants (Yang et al., 2024). The bottleneck in these protocols lies in the *in vitro* regeneration from RNP transfected protoplasts. Examples of regeneration from CRISPR-edited potato and tomato protoplasts have been reported respectively by Andersson et al. (2018) and Liu et al. (2022), but low efficiency in both transfection and regeneration steps still hamper the application of this technique to other *Solanum* species, such as bell pepper (Choi et al., 2024; Kim et al., 2020) and eggplant.

Recently, a protocol for isolation and PEG mediated transfection of eggplant protoplasts with a vector expressing yellow fluorescent protein (YFP) was published (Wang et al., 2022), but no evidence of CRISPR/Cas9 mediated genome editing in eggplant protoplasts can be found in the literature.

In this work, we aim at developing an efficient protocol for protoplast isolation and regeneration from eggplant cotyledonary leaves. Furthermore, by comparing two different delivery approaches, PEG and lipofection-mediated transfections, we intend to evaluate the feasibility of RNP-mediated transfection in this species. As target gene we selected *SmChl_H*, which encodes the H subunit of the magnesium protoporphyrin chelatase (Walker and Willows, 1997), an enzyme involved in chlorophyll biosynthesis. Reduction or absence of this enzyme results in yellow-coloured leaves, caused by a reduction in chlorophyll synthesis, as previously demonstrated through Virus-Induced Gene Silencing in eggplant (H. Liu et al., 2012) and other *Solanaceae* species (Hiriart et al., 2002; Ryu et al., 2004). By optimising previously published protocols for isolation and regeneration of protoplasts from other species of the *Solanaceae* family, in the present work we achieved efficient isolation, callus regeneration and genome editing of eggplant protoplasts. We also investigated the editing outputs in regenerated calli and took a deeper look at the parameters that can influence them.

## Materials and methods

### Plant material and *in vitro* culture conditions

*S. melongena* cultivar ‘Black beauty’ was used in this work. Seeds were surface sterilized by soaking them for 30 sec in 70% ethanol followed by 40% sodium hypochlorite solution for 20 min. They were then rinsed 3 times with sterile distilled water and left overnight in water at RT in the dark. To improve seed germination, the seeds were soaked for an additional day in a solution containing 750 mg/l GA3 and finally placed on sterile germination medium (2.4 g/l MS + vitamins, 15 g/l sucrose, 8 g/l plant agar) in plastic boxes. The plant germination took place at 25 °C in the dark for a week before being transferred to a day/night cycle of 16/8 h (light intensity of 300 µmol m^-2^ s^-1^ PPFD) for growth. After 3 weeks, fully grown cotyledonary leaves were ready to be used for protoplast isolation.

### Protoplast isolation

Protoplast isolation was performed as previously described in potato (Nicolia et al., 2021b) with modifications aiming at enhancing plant tissue digestion to improve protoplast yield. The components of Medium B (conditioning medium), C (enzyme solution), E (culture medium), and all the solutions used during the isolation steps and subsequent culture of protoplasts (plasmolysis solution, wash solution, sucrose solution transformation buffer 1 and 2, PEG solution, alginate solution, floating solution, setting agar, release medium) can be found in Nicolia et al. (2021b). Briefly, the following modifications were applied: ~30 cotyledonary leaves (1-1,5 g) from 3- week-old plantlets were used for protoplast isolation. The cotyledonary leaves were sliced and treated with enzyme solution (medium C) at 25 °C for 20 hours. After this incubation, an additional step of 2 hours of shaking (50 rpm) at RT was used to allow protoplasts to be released from the tissues. Protoplasts were collected after centrifugation and purification on a sucrose gradient, and the yield was quantified using a haemocytometer under optical microscope.

The optimal time for efficient enzymatic digestion was determined by incubating a same number of cotyledonary leaves in Medium C for 14, 17 and 20 hours at 25 °C. The incubation time providing the highest yield was selected for further experiments (**Supplementary Table 1**).

Protoplasts viability was assessed with 4% w/v Trypan Blue staining, calculating the % viability as the number of observed protoplasts that are not stained blue/number of total protoplasts observed × 100.

### gRNAs design and efficiency analyses

Three different gRNAs were designed with the online CRISPOR tool (http://crispor.tefor.net/) targeting *SmChl_H* gene (SMEL4.1_11g022140.1.01). They were chosen on the basis of their high on-target predicted efficiency and low off-target efficiency, as stated by CRISPOR software (**Supplementary Figure 1A**).

Secondary structure prediction of the gRNAs was carried out with RNAfold WebServer (http://rna.tbi.univie.ac.at/cgi-bin/RNAWebSuite/RNAfold.cgi).

All the RNPs components were purchased from Integrated DNA Technologies, Inc. (IDT, Coralville, IA, USA): Alt-R™ S.p. HiFi Cas9 Nuclease, Alt-R^®^ CRISPR-Cas9 tracrRNA, Alt-R^®^ CRISPR-Cas9 crRNA (crRNAs sequences: g5 = TCATACATACAAGGAGCCAA; g8 = GAAAATAGTGTATGTTGTGT; g11 = CTTTTAGTATGTCACAGTTG).

A protocol provided by Integrated DNA Technologies, Inc. was followed to perform an *in vitro* cleavage assay with the three selected gRNAs (i.e. g5, g8 and g11). Briefly, PCR amplification of the *SmChl_H* gene target regions was performed on wild-type DNA of the eggplant cultivar ‘Black Beauty’ using KAPA HIFI Taq (Kapa Biosystems, Boston, USA) with the following PCR program: 1 cycle of 3 min at 95 °C; 35 cycles of 20 sec at 98 °C, 15 sec at 61 °C (g5) or 62 °C (g8-g11), 45 sec at 72 °C; 1 cycle of 1 min at 72 °C. PCR products were purified using the E.Z.N.A.^®^ Cycle Pure Kit following the manufacturer’s instructions. The gRNAs were assembled as follows: crRNAs and tracrRNA were resuspended in duplex buffer to a final concentration of 100 µM, they were mixed in equal amounts and heated at 95 °C for 5 min, then left at RT to cool down and form the gRNA duplex. 10 µl of gRNA duplex (10 µM), 1,6 µl of Cas9 (62 µM stock) and 1 µl 10× PBS buffer (pH 7.4) were mixed with nuclease free water to a 100 µl final volume and incubated at RT for 10 mins to produce RNP complexes. The *in vitro* digestion reaction was performed in a final volume of 10 µl including 1 µl 10× nuclease reaction buffer (200 mM HEPES, 1M NaCl, 50mM MgCl_2_, 1 mM EDTA, pH 6.5), 1 µl RNP (1 µM) and 100 nM DNA substrate from the purified PCR and nuclease free water. The digestion was incubated for 2 hours at 37 °C and the reaction stopped by adding 1 µl proteinase K (20 mg/ml) and incubating at 56 °C for 10 mins. The cleaved product was visualised on a 1,5% w/v agarose gel with a 100-3k bp ladder (GeneRuler 100 bp Plus DNA Ladder, Thermo Scientific).

### RNPs *in vitro* assembly

The gRNAs duplexes were assembled as explained before. Then, 4 µl gRNA duplex (25 µM), 20 µg Cas9 (2 µl of a 62 µM stock) and 1 µl 10× PBS buffer (pH 7.4) were mixed with nuclease-free water to a 10 µl final volume and incubated at RT for 30 mins to allow the assembly of RNPs (1:1.2 gRNA: Cas9 ratio). RNPs were then used for protoplast transfection.

### PEG-mediated transfection with RNPs

Freshly isolated protoplasts were transfected via PEG mediated delivery of RNPs. For each transfection, 10 µl of RNP complexes, assembled as described above, were placed in a 15 ml tube. Then, 100 μl of protoplast suspension (1x10^6^ protoplasts/ml) was added to the same tube and gently mixed before and after adding 110 μl of PEG solution, either 25% (w/v) or 40% (w/v) (Nicolia et al., 2021b). The transfection was stopped by adding 5 ml wash solution in the tube after 3 min (25% PEG) or 30 min (40% PEG).

A negative control treatment was performed by transfecting the same number of protoplasts with Cas9 only (without any gRNAs).

### Lipofectamine-mediated transfection with RNPs

Lipofectamine-mediated transfection was performed following the protocol from Gambino et al. (2024) with some modifications. Two different lipofectamines were used: Lipofectamine™ CRISPRMAX™ Cas9 Transfection Reagent (ThermoFisher Scientific) and Lipofectamine™ 3000 Transfection Reagent (ThermoFisher Scientific). For each transfection reaction, 20 µl of lipofectamine was diluted in 5 µl of wash solution (Nicolia et al., 2021b), mixed and incubated for 10 min at 25 °C, then mixed with RNP complexes assembled as described above and incubated for another 15 min at 25 °C. This reaction mix was combined with 100 μl of protoplast suspension (1x10^6^ protoplasts/ml), mixed with gentle fingertapping and incubated at 25 °C for 1 h and 30 min.

A negative control treatment was performed by transfecting the same number of protoplasts with Cas9 only (without any gRNAs).

### *In vitro* culture of protoplasts and regeneration

Protoplasts embedding in alginate discs and subsequent *in vitro* culture steps were performed following a protocol established for potato (Nicolia et al., 2021b), with the same media composition (medium E, F, G). Five-six weeks after isolation, the calli were generally big enough to be transferred to solid medium. The obtained calli were put on medium E6 from García-Fortea et al. (2020) to induce shoot formation and the media were refreshed every two weeks. Subsequent regeneration steps followed the protocol by Ferrero et al. (2024).

### Molecular screening of edited material

Three months after protoplast isolation and PEG-mediated or lipofectamine-mediated transfection, genomic DNA was extracted from 20 individual calli for each transfection condition using E.Z.N.A.^®^ Plant DNA Kit (Omega Bio-Tek, Norcross, USA) following the manufacturer’s instructions. Primers were designed on the genomic regions flanking the gRNAs to amplify the target sites within the *SmChl_H* locus (g5 forward primer: 5’-AGTATGTCACAGTTGGGGCA-3’; g5 reverse primer: 5’-AACTTTAGCCCCTCTTGCCT-3’; g8-g11 forward primer: 5’-ATGGCTTCTTTGGTTTCTTCAC-3’; g8-g11 reverse primer: 5’-TTCACCCCTTTTAGAGCAGG-3’). PCR amplification was performed using KAPA HIFI Taq (Kapa Biosystems, Boston, USA) with the following PCR program: 1 cycle of 3 min at 95 °C; 35 cycles of 20 sec at 98 °C, 15 sec at 60 °C (g5) or 62 °C (g8-g11), 30 sec (g5) or 45 sec (g8-g11) at 72 °C; 1 cycle of 1 min at 72 °C. After purification with AMPure XP Beads 0,8X (Beckman Coulter), amplicons were sequenced by Sanger method and the knock-out score was estimated for each single callus by analysing the chromatograms using the Synthego ICE online tool (https://ice.synthego.com/). The editing efficiency was calculated as the percentage of edited calli on the total number of calli that underwent Sanger sequencing.

For the molecular screening of the plantlets regenerated from transfected protoplasts, the protocol described above has been followed for DNA extraction from leaves, PCR target amplification and Sanger sequencing analysis.

## Results

### Optimisation of protoplast isolation protocol

**Figure 1:**
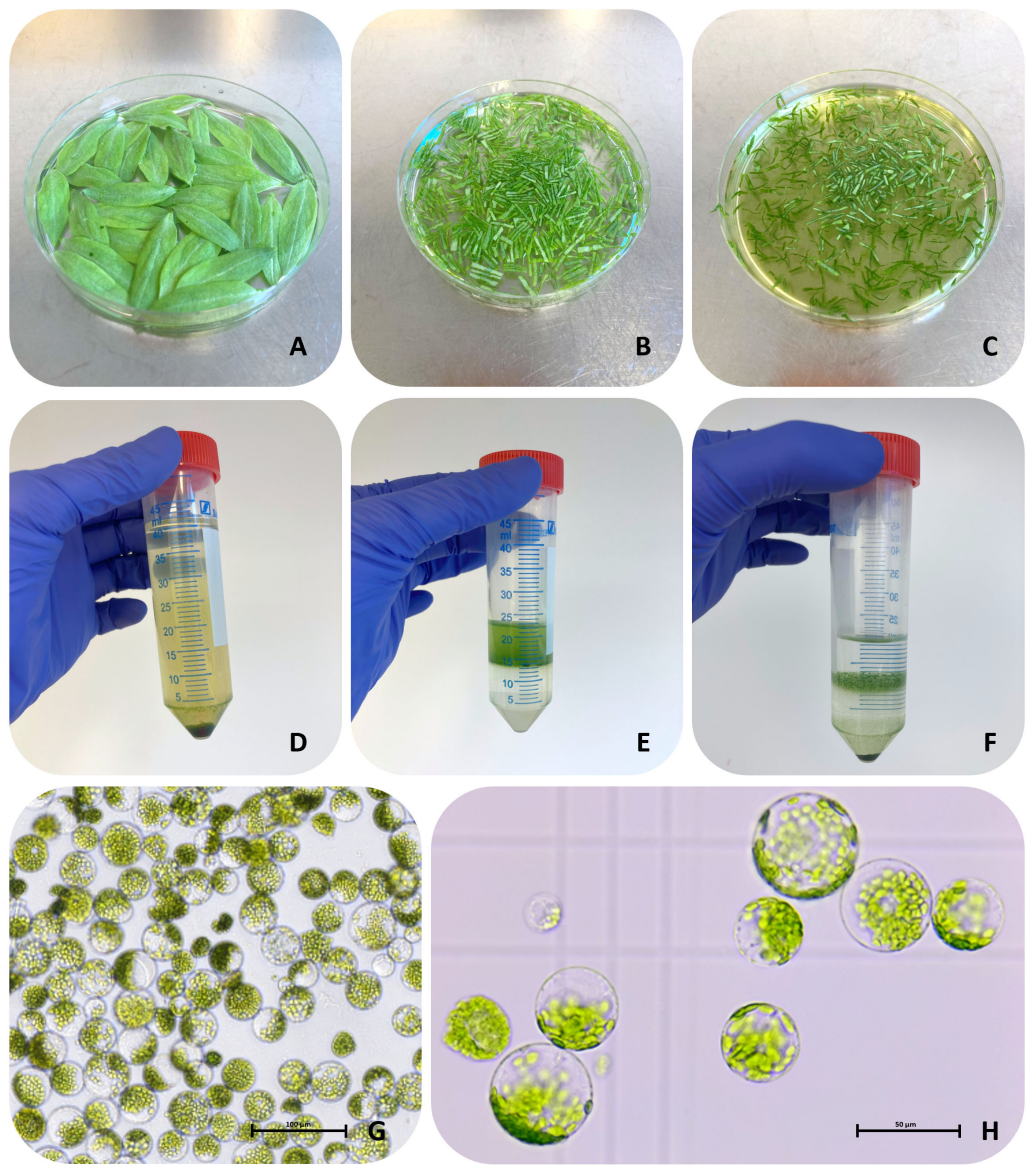
Steps of protoplast isolation protocol: 3-week-old eggplant cotyledonary leaves **(A)**; cotyledonary leaves after being cut in thin slices **(B)**; cotyledonary explants after enzymatic digestion **(C)**; digestion product before centrifugation **(D)**; protoplast purification on sucrose gradient **(E, F)**; optical microscope visualisation of eggplant protoplasts **(G, H)**.

Since very limited knowledge related to eggplant protoplast isolation can be found in literature (Nishio et al., 1987; Sihachakr and Ducreux, 1987; Wang et al., 2022), some improvements are still needed to overcome the difficulties encountered during *in vitro* regeneration. In this view, an existing protocol for potato protoplast isolation (Nicolia et al., 2021b) has been adapted to eggplant in order to meet its specific demands and improve efficiency. In this study, cotyledonary leaves from 3-week-old plantlets were used as starting material (**Figure 1A**), given their higher regeneration potential compared to leaves (Alam and Salimullah, 2021). Cotyledonary leaves were finely sliced (**Figure 1B**) and treated with a digestion solution containing cellulase R10 1% w/v and macerozyme R10 0.2% w/v for 20 hours at 25 °C (**Figure 1C**). Compared to the protocol by Nicolia et al. (2021b), a longer incubation time (20 hours for eggplant cotyledonary leaves vs 14 hours for potato leaves) was needed for an efficient digestion, coherently with eggplant tissues being thicker if compared to potato leaves. The optimal time required for efficient digestion of the cell wall was determined by counting the protoplasts isolated after 14, 17 and 20 hours of digestion, revealing that the longest incubation time provided the higher number of protoplasts (above 1.5x10^5^/ml), compared to 14 hours (8. 5x10^4^/ml) and 17 hours (1x10^5^/ml) (**Supplementary Table S1**).

After centrifugation (**Figure 1D**) and purification on a sucrose gradient (**Figures 1E, F**), viable protoplasts were retrieved (**Figures 1G, H**). The number of isolated protoplasts ranged from 1.8x10^5^/ml to 4.5x10^5^/ml depending on the quality of the digested tissue, and the vast majority of isolated protoplasts appeared intact under the light microscope. The average viability, as assessed with Trypan Blue staining, resulted generally high (92 ± 1%). The isolation yield of this protocol was lower than the one described by Wang et al. (2022), but the milder and more gentle treatment, as proposed here, may help to enhance the protoplasts’ ability to regenerate *in vitro*.

### *In vitro* regeneration from eggplant protoplasts

**Figure 2:**
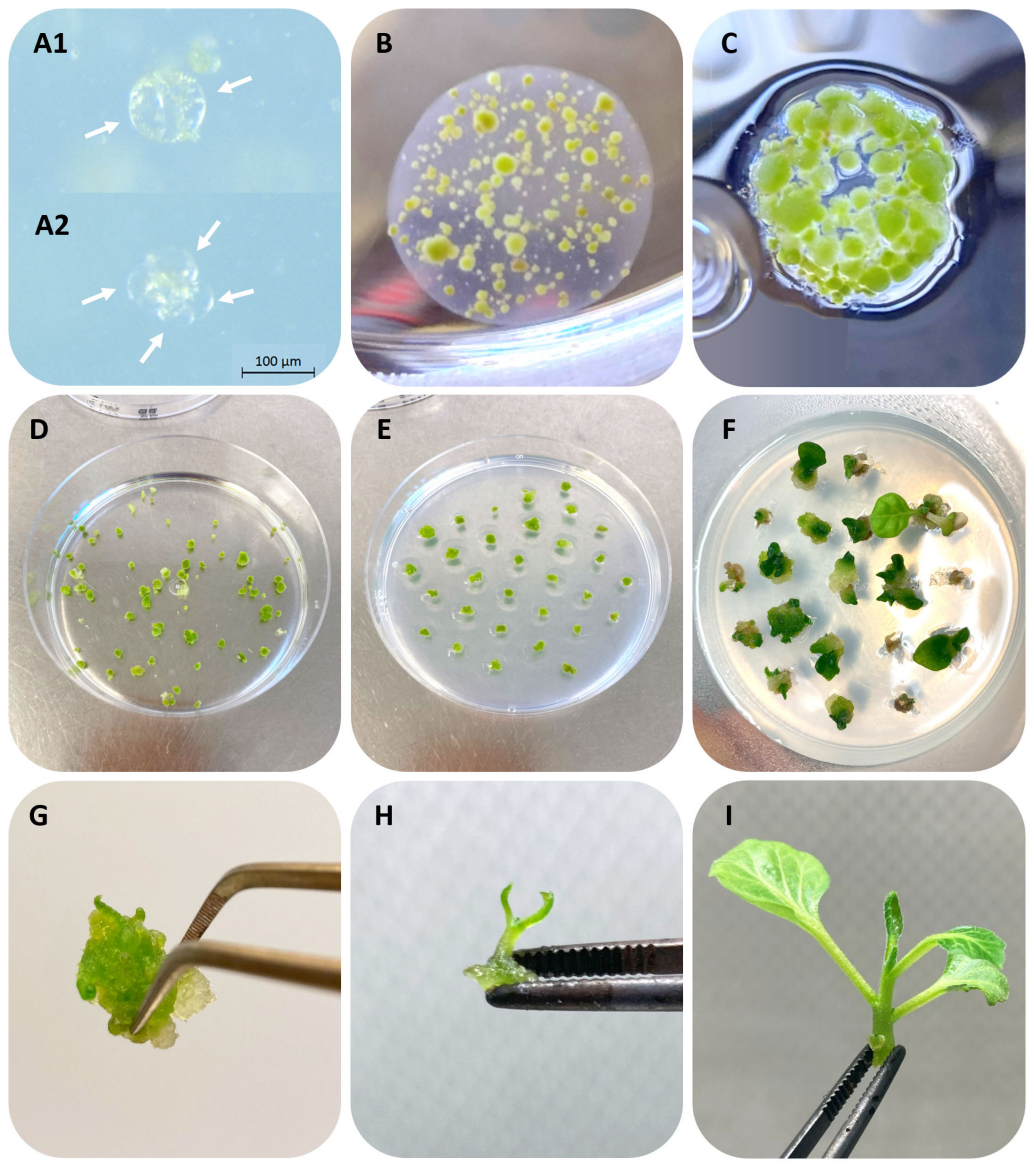
Steps of *in vitro* regeneration from protoplasts: first cell division **(A1)**; second cell division **(A2)**; microcalli after 3 weeks of culture **(B)**; calli growing inside the alginate disc **(C)**; calli released from alginate discs **(D)**; calli on solid medium **(E)**; calli developing first shoots **(F)**; early stage of caulogenesis **(G)**; shoots developing from calli **(H)**; whole regenerated plant **(I)**.

After isolation, 1x10^5^ protoplasts were embedded in alginate discs to avoid physical damage and promote callus development from single cells. Five to seven days after isolation, the first cell divisions were visible under the microscope (**Figure 2A**) and the progressive growth of microcalli took place inside the alginate discs until the calli were big enough to be visible with the naked eye, about 3 weeks later (**Figure 2B**). When the calli filled up the disc (**Figure 2C**), they were released from the alginate (**Figure 2D**) and directly moved onto solid medium about 6 weeks after isolation (**Figure 2E**). The same medium that previously proved to be effective for *in vitro* regeneration from eggplant cotyledonary explants (Ferrero et al., 2024; García-Fortea et al., 2020) proved to be suitable for regeneration from protoplasts as well, stimulating the development of shoots and leaves from calli (**Figures 2F, G, H**). The first whole plants were obtained 4–5 months after isolation (**Figure 2I**) and were moved to soil for acclimatisation.

The number of calli obtained from the same starting number of protoplasts was highly variable and dependent on the transfection method (**Table 1**). The concentration of PEG used for transfection greatly influences regeneration, as suggested by protoplasts treated with 25% PEG yielding 11 times more calli compared to the 40% PEG treatment (**Table 1**). Moreover, different formulations of lipofectamines also had an impact on regeneration: transfection with the same concentrations of Lipofectamine CRISPRMAX™ provided 11 times more calli than Lipofectamine™ 3000 mediated treatment (**Table 1**). The number of calli obtained from untreated controls was as expected higher than any of the treatments, yielding 34% more calli when compared to the best regeneration condition with 25% PEG (**Table 1**). In fact, as reported in other works, PEG can negatively affect regeneration because of its cytotoxicity on protoplasts (Gambino et al., 2024; Masani et al., 2014).

**Table 1:** Number of regenerated calli, starting from the same number of protoplasts (1x10^5^), from different transfection protocols (PEG vs lipofection) divided on the basis of the used gRNA, and untransformed controls (CTR).

Treatment		gRNA5	gRNA8	gRNA11	Total
PEG	25%	420	93	108	621
	40%	36	11	9	56
Lipofectamines	Lipofectamine™ 3000	2	38	6	36
	CRISPRMAX™	107	256	167	530
CTR	941				941

### Genome editing in eggplant protoplasts: PEG and lipofectamine mediated delivery of RNPs

It is well known that editing efficiency (EE) can be affected by many factors in the transfection protocols, so we aimed at assaying different methods. We chose two concentrations of PEG (i.e. 25% and 40%) and two formulations of lipofectamines (i.e. Lipofectamine CRISPRMAX™ and Lipofectamine™ 3000) to evaluate the efficiency of different methods, both in terms of EE and regeneration, which should be optimised. Lipofection is a new method for RNP delivery into plant cells, and it is expected to have a milder impact on the cells’ ability to regenerate because it doesn’t involve creation of pores in the membrane, unlike PEG mediated transfection. We designed three different gRNAs (i.e. gRNA5, gRNA8 and gRNA11) on the *SmChl_H* gene in order to evaluate different targets.

*In silico* analyses (**Supplementary Figure 1A**) and secondary structure modelling (**Supplementary Figure 1B**) suggested high editing efficiency for all the three chosen gRNAs and also an *in vitro* cleavage assay confirmed this hypothesis (**Supplementary Figure 1C**). We therefore used the three of them for protoplast transfection with RNPs, in order to evaluate their efficiency *in vivo*.

The EE, hereafter calculated as the percentage of edited calli on the total of calli that underwent sequencing, was evaluated in all the tested conditions through analysis of Sanger chromatograms (**Table 2**). 25% PEG was the transfection protocol that provided the highest EE, with 59% of the analysed calli being edited in at least one of the alleles. The EE for 40% PEG treatment resulted slightly lower, with 44% of the calli that underwent sequencing showing editing on at least one allele. Lipofectamine-mediated transfection gave much lower EE if compared to PEG treatments, with 11% for Lipofectamine™ 3000 and 2% for Lipofectamine CRISPRMAX™ (**Table 2**).

**Table 2:** Editing efficiency, calculated as the percentage of edited calli on the total number of analysed calli, divided by transfection method (i.e. 25% PEG, 40% PEG, Lipofectamine™ CRISPRMAX™ and Lipofectamine™ 3000), gRNA used (i.e. gRNA5, gRNA8 and gRNA11) and phenotype (green or chlorotic).

Treatment	total EE	EE gRNA5	EE gRNA8	EE gRNA11	EE green	EE chlorotic
**25% PEG**	59%	59%	61%	77%	29%	82%
**40% PEG**	44%	50%	16%	66%		
**Lipofectamine™** 3000	11%					
**CRISPRMAX™**	2%					

Considering the remarkably higher percentage of edited calli obtained in PEG treatments, they were further analysed in detail to better evaluate the impact of selected parameters (% of PEG, gRNA choice).

The recorded EE in all the PEG-mediated treatments is summarised in **Table 2**. The highest EE was obtained on gRNA11 with 25% PEG treatment (77%), and lower values were observed for the other gRNAs under the same experimental condition (59% for gRNA5 and 61% for gRNA8). Transfection with 40% PEG yielded the highest EE on gRNA11 (66%) and EE of 50% and 16% for gRNA5 and gRNA8 respectively. These results clearly demonstrate that a lower percentage of PEG used in the transfection provides a higher number of regenerated calli (**Table 1**) without compromising the editing output, considering that the resulting EE is higher than in all the other treatments (**Table 2**). This observation is consistent with the demonstrated cytotoxicity of PEG, that makes a lower percentage of PEG a suitable choice for eggplant protoplasts transfection.

**Figure 3:**
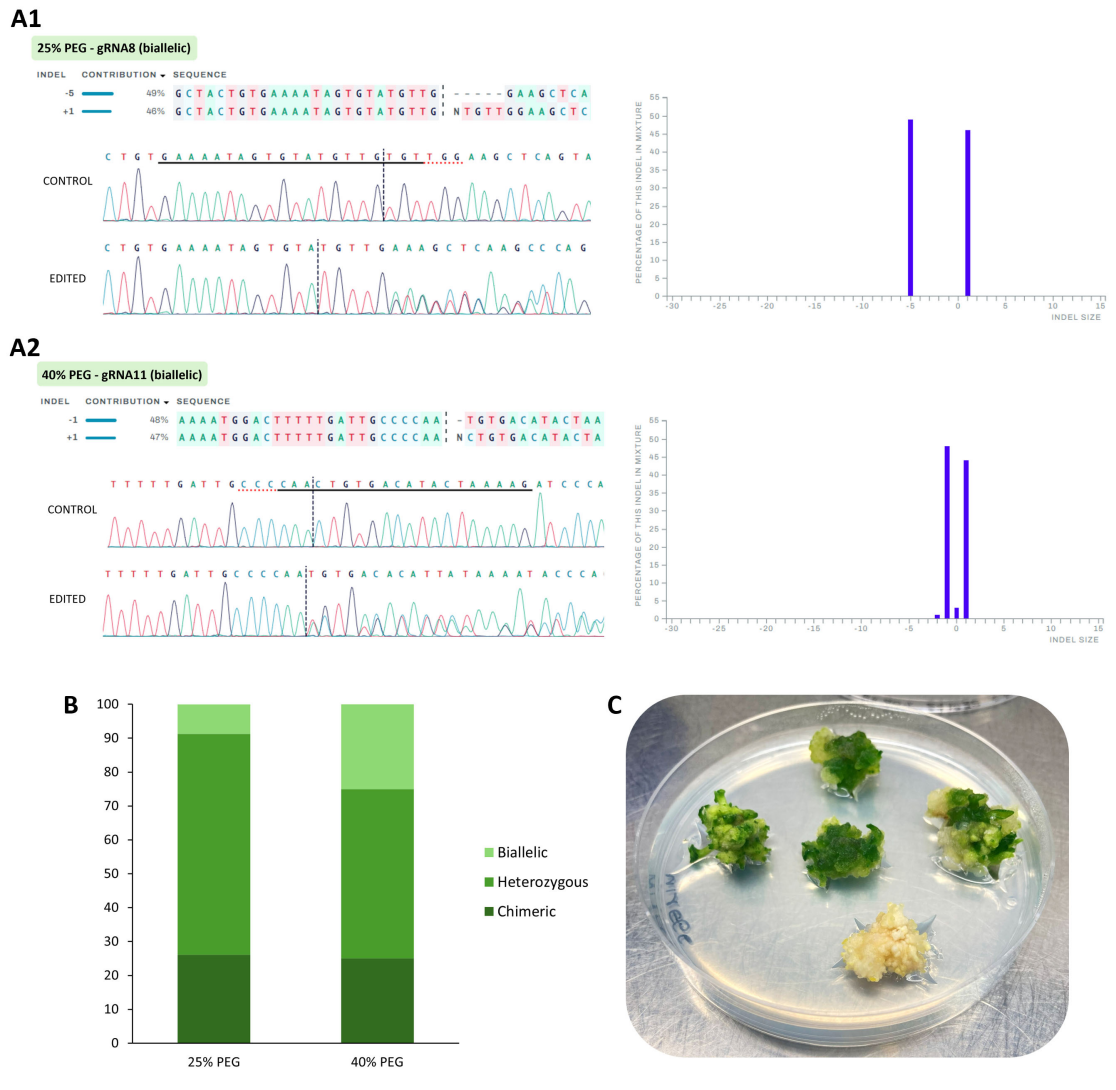
Analyses on editing efficiency in PEG mediated transfections. **(A1-2)** Output of Sanger sequencing analysis of two biallelic edited calli through Synthego software (https://ice.synthego.com/#/). Relative contribution of each allele present in the edited population is reported along with chromatogram alignment between edited and control samples and an indel plot. **(B)** Allelic status of edited calli, represented as a percentage of biallelic, heterozygous or chimeric calli on the total of calli that underwent sequencing. **(C)** Chlorotic vs green phenotype in regenerated calli.

Going into further details, the output of Sanger sequencing on individual calli was analysed to elucidate the allelic status after editing and regeneration from protoplasts (**Figure 3B**). Approximately half of the edited calli (in particular, 65% and 50% of the calli coming from 25% PEG and 40% PEG treatments, respectively) showed a heterozygous allelic asset, with only one of the two alleles carrying indels. A smaller percentage of the analysed calli resulted biallelic, meaning that both the alleles were edited but with different mutations, representing 9% and 25% of the analysed calli coming from 25% PEG and 40% PEG, respectively. Two examples of chromatograms coming from sequencing of biallelic calli are reported in **Figure 3A**. The remaining calli (26% for 25% PEG treatment and 25% for 40% PEG treatment) showed chimerism (harbouring more than two alleles), with editing outputs that do not fall under the above-mentioned categories, probably due to the development of one callus from more than one protoplast.

Another parameter that may greatly influence editing efficiency is the choice of gRNA. Although many computational tools have been developed in recent years to support the selection of target regions that can be efficiently edited, it is still difficult to design the optimal gRNA (Chen and Wang, 2022). In this work, we selected three gRNAs targeting the first exon of *SmChl_H* gene, all of them having high on-target and low off-target scores given by the online prediction tool (**Supplementary Figure 1A**), and named them gRNA5, gRNA8 and gRNA11. All the chosen gRNAs had a correct secondary structure (**Supplementary Figure 1B**) and proved to be effective in an *in vitro* cleavage assay. In the latter experiment, gRNA11 resulted particularly efficient if compared to the two other gRNAs. The average editing efficiency for each gRNA was calculated regardless of the PEG percentage used, in order to clarify the importance of gRNA choice (**Table 2**). The results showed that gRNA8 caused a lower EE (40%) if compared to gRNA5 (56%) and gRNA11, that gave the highest EE (70%), consistently with what was observed in the cleavage assay *in vitro*.

The knockout of *SmChl_H* gene causes a reduction in chlorophyll biosynthesis that ultimately leads to a chlorotic phenotype in edited tissues, appearing whitish or yellowish (**Figure 3C**). We analysed the editing efficiency in relation to the observed phenotype in regenerated calli and it resulted that the 82% of chlorotic calli was edited, while only the 29% of green calli showed mutations (Tablr 2). Indeed, fully edited biallelic calli were retrieved only among the chlorotic ones. These data demonstrate that editing on *SmChl_H* creates a reliable visual system that can support the screening of edited mutants from the early stages of regeneration.

Even though great difficulties were encountered in further steps of *in vitro* regeneration from eggplant calli, three plantlets were obtained from the 25% PEG treatment, and they were acclimatised in soil pots. Despite a normal green phenotype, target sequencing of *SmChl_H* gene revealed a heterozygous mutation on gRNA5 in one of them (**Supplementary Figure 2**) providing the first edited plant regenerated from eggplant protoplasts.

## Discussion

New genomic techniques (NGTs), often defined as precision breeding, are breeding techniques that allow targeted modification of genomes in a faster and more precise way if compared to conventional breeding methods. The current European GMO legislation is characterized by a strong precautionary approach and requires authorization for cultivation (Directive 2001/18/EC; European Parliament, 2001) and for the use as food and feed (Regulation 1829/2003/EC; European Parliament, 2003) (Zimny, 2022) of genetically modified organisms, currently including those plants developed with NGTs. The European Union is now in the process of drafting and implementing a new regulatory framework for NGTs to support the production of new plant varieties, meeting the agriculture’s requirements. Most arguments for a new, less strict regulation for NGT plants claim that these techniques produce modifications that in principle could have been achieved through conventional breeding or classical mutagenesis, suggesting that if no foreign DNA is being introduced in the genomes, NGT plants could be suitable for an exemption from the GMO Directive (Habets and Macnaghten, 2024). After a long trialogue, in December 2025 the European Council reached a provisional agreement with the Parliament and the Commission on the legal framework for NGTs, that would define the category 1 plants as conventional-like (Council of the EU, 2025; European Commission. Directorate-General for Health and Food Safety, 2025). At the end of January 2026, the European Parliament’s Committee on Environment, Public Health and Food Safety (ENVI Committee) voted to approve a deal on NGTs. On June 17–2026 the European Parliament gave its final approval to the regulation on plants developed using new genomic techniques (NGTs), bringing the EU legislative process to a close.

Genome modifications falling into NGT category 1 can be obtained through CRISPR/Cas mediated stable transformation through *A. tumefaciens*, but time-consuming crossing steps are needed to segregate transgenes and obtain transgene-free edited plants. This is not feasible for plants with a high heterozygosity that are vegetatively propagated to maintain genetic integrity. A possible solution to this would be to use a method that doesn’t involve stable integration of exogenous DNA in the genome, thus avoiding genetic segregation steps. From this perspective, RNP mediated transfection of protoplasts allows the introduction directly into naked cells of the Cas9-gRNA complex, that will be degraded by the cell right after its action, leaving no trace in the genome. This system has been successfully applied to many plant species, including Solanaceous crops like tomato (Kang et al., 2024; Y. Liu et al., 2022; Nicolia et al., 2021b) and potato (Andersson et al., 2018; González et al., 2020; Moon et al., 2022; Zhao et al., 2021). Some preliminary editing results have also been obtained in a recalcitrant species like bell pepper but without regeneration (Choi et al., 2024; Kim et al., 2020; J.-H. Park and Kim, 2023).

Eggplant is a crop species with high economic importance, but the application of genome editing tools to this species is still hampered by low editing efficiencies, regeneration protocols that are highly cultivar specific, and long regeneration times that make it difficult to obtain transgene-free edited eggplants. In fact, Ferrero et al. (2024) reported low regeneration and editing efficiencies in *Smdmr6–1* eggplant mutants developed by stable transformation, and three self-pollination steps were required to fix the mutation in homozygosis and to segregate the transformation cassette. Maioli et al. (2020) developed edited plants through stable transformation, but they were not able to obtain a *Cas9*-free eggplant line with simultaneous knockout of *SmPPO4-5–6* genes. Recently, Wang et al. (2022) published a protocol for protoplast isolation from several Asian eggplant cultivars and also demonstrated PEG mediated transfection of eggplant protoplasts with a vector expressing YFP. However, RNPs mediated genome editing and protoplast regeneration in eggplant still need to be evaluated, which represent the main goals of the present work.

Our protocol was developed starting from a well-established protocol for protoplast isolation from potato leaves (Nicolia et al., 2021a) that had additionally been optimised for tomato cotyledonary leaves (Y. Liu et al., 2022). Here, we slightly changed the digestion conditions to adjust it for eggplant cotyledonary leaves. This was achieved by increasing the incubation time, which resulted in a large number of isolated protoplasts apparently intact as observed under the microscope. The number of protoplasts obtained from eggplant tissues (2x10^5^ – 5x10^5^ per gram of fresh weight) was slightly lower than those of potato (7.6x10^5^ – 1.9x10^6^ per gram of fresh weight; Nicolia et al., 2015) and tomato (2x10^5^ – 2x10^6^ per gram of fresh weight; Y. Liu et al., 2022), and much lower than the number reported by Wang et al. (2022) in eggplant (1.2x10^7^ per gram of fresh weight). This could be due to differences in the composition of the starting tissue or to the adoption of a milder digestion treatment that yields less protoplasts but could then, on the other hand, result in protoplasts more prone to regeneration. First cell divisions and microcalli development occurred at approximately the same time after isolation as for potato and tomato (Y. Liu et al., 2022; Nicolia et al., 2015). After releasing the calli from alginate discs, the regeneration protocol had to be adapted to the species. We followed the same protocol that was successful for *in vitro* regeneration from eggplant cotyledonary explants (Ferrero et al., 2024; García-Fortea et al., 2020), and callus growth was observed. Further regeneration steps were slower and more difficult than for potato and tomato due to strong vitrification processes, although some plants were obtained 4–5 months after isolation. However, additional optimisation may be needed to increment the shoot regeneration rate from eggplant protoplasts.

The protocol we applied for PEG mediated transfection was based on what has been published for potato (Andersson et al., 2018; Nicolia et al., 2021a) with modifications, such as the gRNA: Cas9 ratio and the quantity of Cas9 used in each transfection. This way we were able to make the editing efficient, as confirmed by editing efficiencies around 60% for 25% PEG treatment and above 40% for 40% PEG treatment (**Table 2**). These data are similar to what Nicolia et al. (2021b) found in tomato calli, that showed a percentage of edited calli ranging from 30% to 90%, depending on the target gene. In this work, we also investigated the influence of the PEG percentage used in the transfection step on editing and regeneration. We tested two different conditions, 25% and 40% PEG, with different incubation times (3 or 30 minutes respectively) and evaluated the number of regenerated calli and the editing efficiency. 25% PEG treatment yielded 11 times more regenerated calli compared to 40% PEG treatment (**Table 1**), coherently with the proven cytotoxicity of PEG (Biswas et al., 2022; Gambino et al., 2024; Mahmoud et al., 2022) which causes lower viability of protoplasts linked to higher PEG concentration. Moreover, analysing the allelic asset of individual calli after sequencing, highlighted that heterozygous and biallelic mutational status, *i.e.* one or two alleles are edited, respectively, represent the vast majority of the edited calli (**Figure 3B**), confirming the high efficiency of RNP mediated transfection obtained. In particular, biallelic calli are clearly the most interesting outcome, as they could potentially develop fully edited transgene-free plants directly eligible for NGT category 1.

In addition, we tested another approach to deliver editing reagents into cells called lipofection, which enables the uptake of RNPs thanks to cationic lipids. This technique was successfully applied for genome editing in lettuce (J. Park et al., 2019), tobacco (W. Liu et al., 2020), citrus (Mahmoud et al., 2022) and grapevine (Gambino et al., 2024) protoplasts, obtaining improved *in vitro* regeneration from recalcitrant cultivars. We followed the protocol published for grapevine (Gambino et al., 2024) with minor modifications to optimise regeneration, and we tested two different lipofectamine formulations (i.e. Lipofectamine CRISPRMAX™ and Lipofectamine™ 3000) for RNPs transfection. From this experiment we obtained a number of regenerated calli that was similar to the PEG experiment: in particular Lipofectamine CRISPRMAX™ and Lipofectamine™ 3000 provided a regeneration rate slightly lower than 25% PEG and similar to 40% PEG, respectively. The editing efficiency for eggplant ranged from 2% to 11% depending on the lipofectamine formulation, which was much lower than the percentage obtained in PEG mediated transfections. Nevertheless, the percentage of edited plants was 11.3% in grapevine (Gambino et al., 2024) and 6% in tobacco (W. Liu et al., 2020), which is in line with our data. Regarding *in vitro* regeneration, we didn’t observe a significant difference in the number of regenerated calli comparing PEG and lipofectamine mediated transfection (**Table 1**), in contrast to the experiment in grapevine.

Another aspect that could be considered in our work is the suitability of *Chl_H* as a visual system for the early-stage identification of mutants. Full knockout of the *Chl_H* gene confers a chlorotic phenotype to the edited tissues due to a deficiency in chlorophyll biosynthesis, making them recognisable already at callus stage and convenient for screening. However, a total lack of this gene’s functionality may impact on the ability of the calli to develop correctly because of a total lack of chlorophyll, potentially explaining a lower regeneration rate from fully edited tissues. One fully developed plant with heterozygous editing was obtained. Although it did not show the chlorotic phenotype as one allele was still functional, it demonstrates that the development of our editing protocol, combined with the regeneration protocol setup, represents a promising starting point. The *in vitro* regeneration protocol needs to be further improved in order to obtain a better development of plants from edited calli, and a further optimisation of the lipofection protocol could be explored, using a different target gene for gene editing.

The present work represents the first example of genome editing in eggplant protoplasts, enabling the obtainment of transgene-free edited eggplant lines that could ultimately meet the legislative requirements and be suitable for NGT category 1, which would represent the ultimate objective of our research.

## Data Availability

The raw data supporting the conclusions of this article will be made available by the authors, without undue reservation.
