# Supplementary material for: Tiny but powerful: protoplast isolation in Solanum melongena L. and RNPs mediated genome editing
Source: Front Plant Sci. 2026 Jul 16;17:1846175. doi: 10.3389/fpls.2026.1846175 (PMC13422435; doi:10.3389/fpls.2026.1846175)
Supplement: Supplementary file 1 [file DataSheet1.docx]

**SUPPLEMENTARY MATERIAL**

**
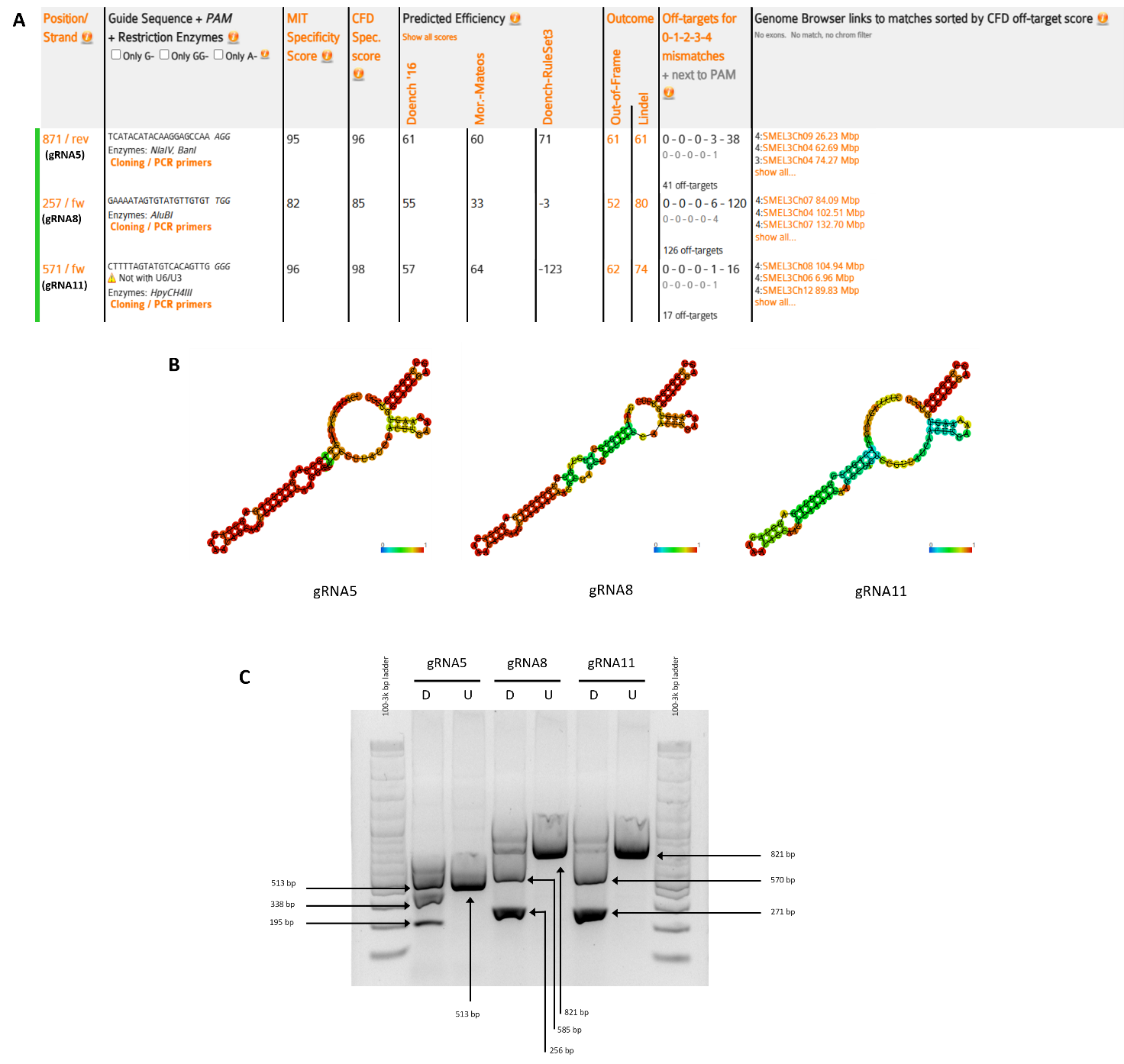
**

**Figure S1** - *In silico* and *in vitro* analyses of gRNA efficiency. (**A**) Output from CRISPOR software (http://crispor.tefor.net/) for the three selected gRNAs providing on-target efficiency scores and off-target analysis. (**B**) Secondary structure of the three selected gRNAs as reported by RNAfold WebServer (http://rna.tbi.univie.ac.at/cgi-bin/RNAWebSuite/RNAfold.cgi). Minimum free energy structure drawing encoding base-pair probabilities (0-1). Results have been computed using RNAfold 2.6.3. (**C**) *In vitro* cleavage assay of the three selected gRNAs. From left to right: gRNA5 digested (D) and undigested (U), gRNA8 digested (D) and undigested (U) and gRNA11 digested (D) and undigested (U). The amplicon size in bp is reported near black arrows. 100-3k bp ladder is used as reference for amplicon length.

**

**

**Figure S2** - Output of Sanger sequencing analysis of the edited plant regenerated from transfected protoplasts through Synthego software (https://ice.synthego.com/#/). Relative contribution of each allele present in the edited population is reported along with chromatogram alignment between edited and control samples and an indel plot.

| Digestion solution | Temperature | Time | Yield | Selected |
| --- | --- | --- | --- | --- |
| cellulase R10 1% w/v + macerozyme R10 0.2% w/v | 25°C | 14 h | 8. 5x10^4^/ml | No |
|  |  | 17 h | 1x10^5^/ml | No |
|  |  | 20 h | > 1.5x10^5^/ml | Yes |

**Table S1** - Summary of digestion conditions tested for protoplasts isolation from 30 cotyledonary leaves.
